# An intriguing vision for transatlantic collaborative health data use and artificial intelligence development

**DOI:** 10.1038/s41746-024-01005-y

**Published:** 2024-01-23

**Authors:** Daniel C. Baumgart

**Affiliations:** Precision Health Signature Area, College of Health Sciences, College of Natural and Applied Sciences all at University of Alberta, Edmonton, Alberta Canada

**Keywords:** Health care

## Abstract

Our traditional approach to diagnosis, prognosis, and treatment, can no longer process and transform the enormous volume of information into therapeutic success, innovative discovery, and health economic performance. Precision health, i.e., the right treatment, for the right person, at the right time in the right place, is enabled through a learning health system, in which medicine and multidisciplinary science, economic viability, diverse culture, and empowered patient’s preferences are digitally integrated and conceptually aligned for continuous improvement and maintenance of health, wellbeing, and equity. Artificial intelligence (AI) has been successfully evaluated in risk stratification, accurate diagnosis, and treatment allocation, and to prevent health disparities. There is one caveat though: dependable AI models need to be trained on population-representative, large and deep data sets by multidisciplinary and multinational teams to avoid developer, statistical and social bias. Such applications and models can neither be created nor validated with data at the country, let alone institutional level and require a new dimension of collaboration, a cultural change with the establishment of trust in a precompetitive space. The Data for Health (#DFH23) conference in Berlin and the Follow-Up Workshop at Harvard University in Boston hosted a representative group of stakeholders in society, academia, industry, and government. With the momentum #DFH23 created, the European Health Data Space (EHDS) as a solid and safe foundation for consented collaborative health data use and the G7 Hiroshima AI process in place, we call on citizens and their governments to fully support digital transformation of medicine, research and innovation including AI.

## Introduction

Traditionally, patients present to healthcare systems with specific symptoms or through a preventive healthcare program. Healthcare providers then capture information from the patient’s medical history, perform a physical exam, review clinical data, order additional investigations, before eventually arriving at a diagnosis, prognosis and therapy based on their own medical knowledge, clinical experience and a thought and decision process. As the case evolves, decisions are critically revisited and adjusted. Over time healthcare professionals *learn* from their intervention outcomes and increasingly gather experience *informing* their *future decisions*.

Medical knowledge and data are growing exponentially. The body of medical knowledge and clinical experience a healthcare professional can acquire and apply depends on the individual’s medical education and training, motivation, and support provided within their health system. It can multiply and grow faster when working in teams and across disciplines. Ultimately however, effective and efficient medical knowledge acquisition and translation are a function of capacity and time limited by the individual’s life span.

Thus, our traditional approach to diagnosis, prognosis, and treatment, can no longer process and transform the enormous volume of information into therapeutic success, innovative discovery, and health economic performance. The digital revolution in medicine began two decades ago^1^. Advancement of information and communication technology, powerful computer science methodology and digital innovation can help accelerate, enhance and refine medical knowledge acquisition and translation.

Precision health, i.e., the right targeted treatment^2^, for the right person, at the right time in the right place, is enabled through a *learning health system*, in which medicine and multidisciplinary science, economic viability, diverse culture, and empowered patient’s preferences are *digitally integrated* and *conceptually aligned* for *continuous improvement and maintenance of health, wellbeing, and equity*.

Healthcare-embedded research with real-world-evidence^3^ generation may overcome some of the limitations of traditional clinical trials. Artificial intelligence (AI)^4^ including machine learning^5^ has been successfully evaluated in risk stratification, accurate diagnosis and treatment allocation, and to prevent health disparities^6^. Large language models make unstructured data accessible^7^. AI can limit harm in drug development^8^, by deploying computational tools to derive biological insights from large amounts of data, and exploit *“in silico”* computer-assisted structure and binding prediction^9–11^, synthesis^12–15^. and testing well before any trial participant is ever exposed.

Healthcare facilities can become safer^16^ and smarter^17^ through AI enabled patient^18,19^ and staff monitoring^20^, policy enforcement^21^, provider competency assessment^22,23^, and resource allocation^24,25^. Wearables promote continuous, citizen centered, preventive care^26^. Public health threats can be taken on earlier and more effectively^27,28^.

There is one caveat though: dependable AI models need to be trained on population-representative, large and deep data sets by multidisciplinary and multinational teams to avoid developer, statistical and social bias^29^. Moreover, complex diseases can only be understood through *integration*^30^ of basic research and health-related and -defining information (e.g. environmental-, socioeconomic-, infrastructural-, and behavioral-, and social data). Such applications and models *can neither be created nor validated with data at country*, let alone *institutional level* and require a new dimension of collaboration. Federated learning, i.e., use of *decentralized data sources* to build models and transfer learning, enables multiple institutions to collaborate *without changing the physical location of data*. While federated learning still requires a central coordinator, swarm learning^31^ circumvents it by uniting edge computing^32^ and blockchain^33^ enabled asynchronous convergence.

However, other applications require true collaborative data use as envisioned by the European Commission’s European Health Data Space (EHDS)^34^. The EHDS is an ecosystem comprised of rules, standards and practices, infrastructures, and a governance framework empowering individuals through increased digital access to their personal health data, at the national and EU level, support their *free* movement, and foster a genuine single market for electronic health record systems, relevant medical devices, and health AI. It will provide a consistent, trustworthy, and efficient set-up for the use of health data for research, innovation, policy-making and regulatory activities.

Collaborative health data use and AI deployment at scale are disruptive advances that potentially expose us to new risks (i.e., security, data manipulation, misuse, failure to recognize model limitations, and moral ethical dilemmas) that need to be carefully weighed against the above-outlined benefits. Balancing both in medicine and healthcare is perhaps one of the most daunting tasks, as it involves the most sensitive and vulnerable aspects of our lives, but also one where we can have the greatest positive impact at an individual and societal level.

Moral dilemmas for instance arise, when choosing between two AI-enabled accurate, yet equally unfavorable decisions and their consequences^35^. An AI-facilitated therapy recommendation can only address the data-driven aspect of personalized medicine, whereas the individual patient’s choice to accept or reject a therapy recommendation (i.e. choosing a potentially less effective therapy for personal reasons) remains unchanged. Ultimately, the role of both AI algorithms and healthcare professionals remains to help patients (and families) make their own, hopefully increasingly better-informed decisions instead of making those decisions for them.

Ethical challenges do not only result from data access, data ownership and training data quality-related bias. Ethical conflicts anticipated to occur with algorithms may in fact not be rooted in AI itself, but rather exposed by it. Today’s physicians and biomedical researchers find themselves in a conflicted situation to serve their patients, their employers and payors, when other components of the healthcare system may have different interests (i.e. effort & expenditures vs. revenue). These interests determine the goals of AI algorithm development^5^.

The German Federal Ministry of Health in collaboration with the German Aerospace Center (DLR) organized a high-level Data for Health conference^36^ in Berlin, Germany in June and a Follow-Up Workshop^37^ in September at Harvard University in Boston, MA, USA (#DFH23) with a representative group of stakeholders in society, academia, industry, and government along with journalists and both vocal supporters and critics for open and productive discussions. It takes that joint perspective to drive the cultural change needed.

#DFH23 explored various ways of taking the EHDS concept across the Atlantic. Three G7 countries, Germany, the United States and Canada lead the way. The German Federal Ministry of Health is committed to pass legislation to enable collaborative health data use and AI, deploy a national electronic health record aligned with the EHDS, and paperless communication in acute and long-term care. In July the European Commission adopted its adequacy decision for the EU-US Data Privacy Framework, enabling European entities to transfer personal data to participating entities in the US. Government consultations are underway to expand longstanding collaboration in AI with Canada^38^. #DFH23 developed harmonized contractual language and multi-lingual consent documents as a start, realizing many other legal and political challenges remain. Figure 1

**Fig. 1:**
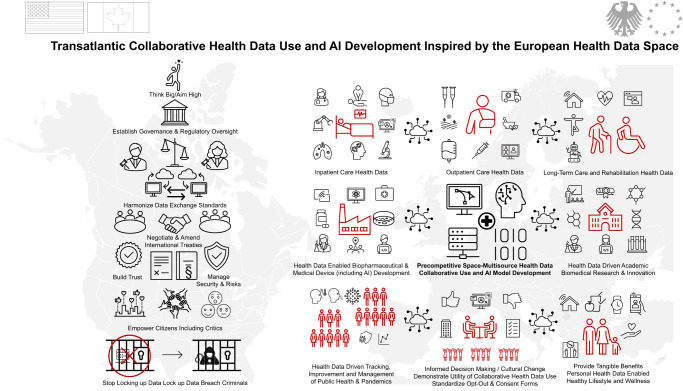
EHDS inspired transatlantic collaborative health data use and artificial intelligence development. Collaborative health data enabled innovation requires cultural change with establishment of trust among all stakeholders in a precompetitive space. Trustworthy communication of goals, risks, benefits and challenges, continuous dialogue with citizens and patients (i.e. joint AI readiness assessment with patients), prejudice free consent and opt-out mechanisms, harmonization of technical and administrative standards (i.e. an international patient summary, harmonized transatlantic patient consent forms) will enable interoperability, aligned regulatory processes (i.e. development of regulatory fact sheets), model use cases and exemplary projects (i.e. a cancer genome tracker, a registry for cancer immune therapy) and a streamlined exchange platform for citizens, researchers, developers and innovators (i.e. a stake holder council, online R&D “tool box”).

Digital inclusion^39^ with engagement of all stakeholders, the reduction of barriers for those with disabilities and the elderly, are critically important for success and have become a social determinant of health. Translation into daily practice critically depends on overcoming the knowledge asymmetry through education and training. To build trust, several model projects with explicit citizen and patient participation demonstrating tangible benefits of collaborative data use and AI to promote healthy living and well-being, advance cancer detection and care, streamline data access, verify AI readiness, accelerate knowledge translation into everyday healthcare were developed and agreed in bar camps, workshops and discussed on the podium at #DFH23.

As the UNs WHO and ITU establish a benchmarking process for AI in health, regulatory bodies need to adapt their workflows^40^. The US FDA decided to regulate AI as a device in line with the International Medical Device Regulators Forum’s commitment to develop a harmonized approach to the management of AI^41^. Transparency (explainable AI [XAI]), decision autonomy level, decision effect size, and opportunity of human oversight and override will be critical for approval. Post marketing surveillance and liability of developers and users are complex tasks since medical, unlike industrial AI models, are not static over their lifecycle^42,43^.

Rising concerns of cyber security with the exploitation of personal data^44^ have led the EU to foster citizens’ right to informational self-determination through the General Data Protection Regulation^45^. Narrow GDPR interpretation may however fail citizens by depriving them of the profound benefits of collaborative data use and AI. #DFH23 attendees propose a legislative and enforcement effort to *prioritize regulating data use* not just protection. Effective legal deterrents rather than unreasonable measures to avoid criminal decryption and re-identification are urgently needed.

In October, one month after the #DFH23 Follow-Up Workshop^37^ at Harvard University the entire G7, i.e. Canada, France, Germany, Italy, Japan, the United Kingdom, the United States as well the EU passed an agreement on International Guiding Principles on Artificial Intelligence (AI)^46^ and a voluntary Code of Conduct^47^ for AI developers referred to as the Hiroshima AI process.

Based on what we know today, it is unlikely that there will be true competition between humans and machines in healthcare compared with the manufacturing or service industries. Although service robots have been experimentally deployed to hospitals and publicly shared places during the COVID-19 pandemic to clean, take temperatures and deliver food, minimize person contacts, this cannot be the primary goal to address manpower challenges or achieve cost-control in healthcare^48^. Human healthcare professionals and machines can supplement each other, as both have unique skills and strengths. In fact, it is conceivable in our competitive, increasingly performance-driven healthcare systems, clinicians will have more time again to spend with their patients, a critical element of empathetic healthcare that has been missed lately due to the economic pressures imposed by hospital operators, payors and governments aiming to lower costs.

With the momentum #DFH23 created, the EHDS as a solid and safe foundation for consented national, EU-wide and transatlantic collaborative health data use as well as the G7 Hiroshima AI process in place, we call on citizens and their governments to fully support digital transformation of medicine, research and innovation including AI implementation. It’s time to rethink healthcare. Our citizens and patients deserve it.

### Reporting summary

Further information on research design is available in the Nature Research Reporting Summary linked to this article.

### Supplementary information

Reporting Summary

## Data Availability

This manuscript does not include any additional data that could be made available.
